# Smart Technologies for Water Resource Management: An Overview

**DOI:** 10.3390/s22166225

**Published:** 2022-08-19

**Authors:** Stefania Anna Palermo, Mario Maiolo, Anna Chiara Brusco, Michele Turco, Behrouz Pirouz, Emilio Greco, Giandomenico Spezzano, Patrizia Piro

**Affiliations:** 1 Department of Civil Engineering, University of Calabria, 87036 Rende, CS, Italy; 2 Department of Environmental Engineering, University of Calabria, 87036 Rende, CS, Italy; 3 CNR-National Research Council of Italy, Institute for High Performance Computing and Networking (ICAR), 87036 Rende, CS, Italy

**Keywords:** smart building, sensors, IoT, water consumption, water level, water leakage detection

## Abstract

The latest progress in information and communication technology (ICT) and the Internet of Things (IoT) have opened up new opportunities for real-time monitoring and controlling of cities’ structures, infrastructures, and services. In this context, smart water management technology provides the data and tools to help users more effectively manage water usage. Data collected with smart water devices are being integrated with building management systems to show how much water is used by occupants as well as to identify the consumption areas to use water more efficiently. By this approach, smart buildings represent an innovative solution that enhances a city’s sustainability and contributes to overcoming environmental challenges due to increasing population and climate change. One of the main challenges is resource-saving and recovery. Water is an all-important need of all living beings, and the concerns of its scarcity impose a transition to innovative and sustainable management starting from the building scale. Thus, this manuscript aims to provide an updated and valuable overview for researchers, consumers, and stakeholders regarding implementing smart and sustainable technologies for water resource management, primarily for building-scale uses.

## 1. Introduction

The increase in population raised human demand, and overuse of water for domestic, agricultural, commercial, and industrial purposes—combined with climate change and pollution—is a serious issue affecting the sustainability of the environment. Since water is a limited natural resource, its proper use and management are crucial [1].

In this context, monitoring water usage in different sectors for better management is one of the aspects that is taken into account in smart city development, which is one of the subjects that has garnered significant interest in the last few years. The development of this innovative concept to improve cities is principally due to the recent progress in information and communication technologies (ICT) and especially the Internet of things (IoT).

Moving towards more intelligent management of the urban environment enhances the quality of human life and increases sustainability. The development of new systems contradicts the background of cyber-physical systems, big data, and digitalization, where processors, sensors, software, communication, and control devices are all integrated to enable informed decisions in an increasingly changing, uncertain, and complex environment. Although a unique definition of this concept is still lacking, a smart city includes many aspects (institutional, technical, social, and economic) in interaction with urban infrastructures [2,3]. Thus, as reported in [4], there are six core areas or aspects that form the concept of smart cities: smart economy (competitiveness); smart governance (citizen participation); smart people (social and human capital); smart mobility (transport and ICT); environment (natural resources); and smart living (quality of life).

All of these features have to be taken into consideration starting from the development of buildings that need to be smart, i.e., able to properly manage resources and provide the best possible comfort to inhabitants. In this regard, the term “intelligent building” was born in the 1980s in the United States to indicate a building with sophisticated devices for management and data network services. More in detail, the definition of building intelligence focused on building automation systems and solutions, known as building management systems (BMS) [5]. Over the past three decades, advances have been remarkable, thanks to technology’s significant evolution, the advent of the Internet, IoT, and hardware and software solutions more generally.

Overall, in a smart building, the optimization of resources takes a key role in reducing their consumption and increasing the sustainability of the building. Thus, sensor devices within smart buildings enable smart water consumption management, early and fast detection of cracks in pipes, and monitoring of water consumption and leakage in individual building segments. Water meters transmit data to a centralized system that stores water consumption information, draws conclusions about possible water leaks, sends alarms to the user or a system, and implements machine learning algorithms with the goal of possible savings and detection of minor and/or consumption trends.

Generally, information on water level, flow, and pressure is crucial in order to identify the best operational management strategy, providing an efficient and cost-effective solution through broader water resource monitoring [6].

Thus far, many reviews and surveys in the literature have analyzed smart water monitoring and management systems. In 2013, Boyle et al. [7] reviewed the devices and global development of intelligent metering for urban water. In another review article [8], the authors presented a survey focused on IoT-based smart water quality monitoring systems specially dedicated to domestic applications. Similarly, Geetha and Gouthami [9] have presented a detailed overview of smart water quality monitoring. While in [10], the authors discussed the architecture and the components of IoT-based systems (water level and water quality monitoring systems). Saad et al. [11] presented a survey on water management and monitoring in agriculture. Recently, Ismail et al. [12] focused on smart IoT-based water management and monitoring system for several applications (agricultural, industrial, and residential). In another study [13], an overview of the water monitoring systems—from traditional techniques to IoT-based monitoring—was carried out by focusing primarily on the IoT-controlled water storage tanks (IoT-WST). Oberascher et al. [2] provided a guideline for communication technologies for monitoring and controlling urban water infrastructures. Vijayan et al. [14] have analyzed basic services in smart buildings (security control, energy management, control and monitoring of HVAC systems, water management, lighting systems, fire detection, health systems, and elderly care) to provide a guideline for future research. Other reviews focused on specific methods for leakage detection and localization in pipelines. In this regard, Adedeji et al. [15] have provided a comprehensive survey of developments in leakage detection and localization methods, while Colombo et al. [16] focused on transient-based leak methods and Mohd Ismail et al. [17] explored vibration detection methods using accelerometer sensors.

Starting from the previous review of studies and surveys and given the key role of these advanced tools in saving water resources, this paper presents an overview of innovative systems to support the widespread use of smart and sustainable technologies for water management. Therefore, the main objective of this manuscript is to provide an update and valuable overview for researchers, consumers, and stakeholders regarding the implementation of smart and sustainable technologies in the field of water resource management. The novelty compared with the other reviews is that this study focuses on smart water monitoring and management primarily at the building scale by investigating IoT-based systems and strategies for measuring water levels, monitoring water consumption, and detecting leaks.

The paper is structured as follows. Section 2 presents the research background on the advent of the Internet of Things (IoT) technologies and an introduction to the smart building concept. Section 3 reviews the sensors used for smart water management—primarily in buildings; focusing on water level, water consumption, and leakage detection sensors. While Section 4 illustrates some sustainable systems for water-saving and management that can be integrated into the smart building concept. Section 5 presents an in-depth discussion about the analyzed systems, current challenges, and future directions of IoT-based smart water monitoring and control systems. Section 6 is left for the work’s main conclusions.

## 2. Research Background

### 2.1. Internet of Things (IoT)

The definition of the “Internet of Things” was first used in 1999 by Kevin Aston, an engineer and researcher at the Massachusetts Institute of Technology in Cambridge. He referred to the IoT as uniquely identifiable interoperable connected objects with radio-frequency identification (RFID) technology [18]. The words “Internet” and “Things” denote an interconnected global network based on sensory, communication, networking, and information processing technologies, which could be the new version of information and communication technology (ICT) [19,20].

IoT devices embedded in these physical objects fall mainly into switches (which send a command to a thing) or sensors (which acquire data and send them elsewhere). This way, information regarding physical elements can be shared, and data collection can be completed through low-cost computing, big data, cloud, analytics, and mobile technologies, with minimal human intervention. Thus, the physical world meets and cooperates with the digital world, creating a hyperconnected world with digital systems that can record, monitor, and regulate every interaction between connected objects [21,22,23].

A typical IoT system functions by creating a continuous feedback loop. Depending on the IoT system type, analysis can be performed by physical intervention or artificial intelligence and machine learning (AI/ML) technologies, either near real-time or long-term. Despite the IoT definition changes depending on different deployment technologies, the foundation of IoT implies that objects in an IoT can be uniquely identified in virtual representations. Within an IoT, all objects can exchange data and, if necessary, process them according to predefined patterns [21,24].

The stages of IoT evolution started with RFID technology, which is increasingly used in logistics, pharmaceutical manufacturing, logistics, and different industries [25,26,27]. Thus, emerging wireless sensor technologies have extended the original IoT concept to ambient intelligence and autonomous control (wireless sensor networks (WSNs), barcodes, smart sensing, RFID, NFC, low-energy wireless communications, and cloud computing) [28,29,30,31]. The evolution of these technologies brings new technologies to the IoT [32,33,34].

The main architecture of IoT includes three layers: the physical layer, the network layer, and the application layer. At the physical layer, sensors collect and transform data from the external environment. Time-sensitive data can be processed simultaneously and collected or stored in the cloud [35]; specifically, data are collected at the network layer and transformed for data processing [36]. Finally, the application layer is responsible for providing specific services to the user.

As reported in [37], the integration of the IoT paradigm into water management processes can provide several benefits—such as improving water management infrastructures by increasing their efficiency, reducing energy cost and human intervention; reducing water management costs; enhancing asset utilization by using sensors and connectivity; and increasing productivity—thereby expanding new and existing business models, based on the three pillars of the IoT (internet-oriented; thing-oriented; knowledge-oriented).

Following the advances in IoT technologies, smart infrastructures are increasingly becoming self-monitored, self-communicated, and especially self-administered. Several factors have enabled this transition, including sustainability, resources management, economics, rapid development of information technology, and advances in computing and communication systems [23].

### 2.2. Smart Building

From its earliest forms, ‘intelligent buildings’—also known as ‘smart buildings’—have had many definitions. As early as 1981, the United Technology Building Systems Corporation of the USA (UTBS Corporation) first used the term “intelligent building”, and two years later, the City Place Building in Hartford, Connecticut, became known worldwide as the first intelligent building [38]. The European Commission has provided a “smart building” specific definition, according to which a smart building includes communication technologies able to link different objects, sensors, and functions, allowing them to communicate with each other and be controlled remotely [39]. Moreover, a smart building can take full advantage of efficiency, realizing resources capable of managing and containing general costs [40]. Based on these definitions, smart building central systems work thanks to digital platforms and electronic sensors. In addition to the timely detection of parameters, these systems can communicate in an automated and integrated way through supervision and control software infrastructure. This type of management makes buildings safer, more efficient, and greener.

Thus, for both private and public use, a smart building is an ecosystem capable of offering different services, divided into six areas: energy, safety, security, comfort, health, and general services [41]. In addition to systems which monitor energy, in a smart building, the control of water resources takes a key role by monitoring consumption and reducing leakage. Thus, several studies—as described in the next section—have investigated the advances in devices and technologies for smart water management.

Overall, the key technical elements of a smart building are (i) building devices and solutions—i.e., technologies for energy generation and efficiency to guarantee occupants’ comfort, safety, and health; (ii) automation technologies—sensors for data collection and actuators to issue commands via control and management platforms; (iii) management and control platforms—software to collect, process and analyze information; (iv) connectivity—communication protocols, wireless or wired, that allow communication between sensors, actuators, and control and the management platform [5,40].

Sensors allow for consumption management, monitoring of system performance, and activation of appropriate interventions to solve equipment malfunctions and prevent alarm triggers. Through this predictive maintenance, in case of an error message, the system collects data to determine the causes and automatically adapts to any problem [41].

Typically, it is possible to identify two main categories of smart building advantages: (i) hard benefits—quantifiable in monetary terms (energy savings, productivity optimization, predictive maintenance); (ii) soft benefits—linked to the improvement of habitation socio-environmental conditions (environmental sustainability, safety, comfort, remote management, remote control, interoperability) [42].

Finally, according to [43], smart buildings have five fundamental features: (1) automation—i.e., the ability to accommodate automatic devices or perform automatic functions (remote detection and control of environmental parameters, remote activation, and switching off of devices); (2) multi-functionality—i.e., the ability to allow the performance of more than one function in a building; (3) interactivity—i.e., the ability to allow interaction among users; (4) adaptability—i.e., the capability to learn, predict, and satisfy the needs of users and mitigate the stresses from the external environment; and (5) efficiency—i.e., the ability to save time and costs.

## 3. Overview of Technologies for Smart Water Management

Water is priceless and vital for every creature; it is a human need necessary for all economic operations. Regardless, the increase in the population is putting the water supply system under severe strain. In this context, smart water management carried out with the application of sensors and telemetry for communication and measurement can be a valuable solution to improve the efficiency of water distribution systems [35,44,45].

The sensors enable real-time monitoring of hydraulic data, automatic monitoring, and alerts from the Cloud platform in case of events such as water leakage or overuse. Smart water systems’ main utility are remote control of valves and pumps [45], which can measure pressure, flow, and consumption [17]. Smart sensors benefit from new intelligent management systems allowed by digital technologies to provide more sustainable and resource-efficient solutions.

Overall, it is possible to include leak detection and leak localization for water consumption monitoring, specifically water loss management, by using noise sensors and accelerometers, popularly used in water distribution infrastructures [17,46]. While regarding pressure measurement, it is possible to use IoT technologies, such as electromagnetic and ultrasonic flow meters and sensors [44,47].

Moreover, the necessity of analyzing vast amounts of data while improving the entire efficiency of systems has directed the attention of the water sector towards advanced digital tools, such as operational digital twins [48]. Digital twins can be realized as combinations of models and real-time data that could present a digital representation of a specific sector in a water system.

A proper water management system optimizes the use of the resource by reducing waste and ensuring sustainable supply. Improving devices and technologies to obtain this purpose is a crucial aspect of preserving water. Thus, it seems that smart water management has to start from the building scale.

Therefore, based on this background, this section presents an overview of the available devices and technologies for smart water resource management, primarily at the building scale.

To carry out this review, Google Scholar, Scopus, Web of Science, and IEE Xplore were considered as research repositories for the identification stage. The first qualification criteria included the following potential keywords and their combination (search strings): “smart sensors”, “smart devices”, “smart water”, “smart building”, “smart home”, “Internet of Things”, “water management”, “water monitoring”, “water level”, “smart tank”, “ultrasonic sensor”, “water flow sensor”, “smart water meter”, “smart monitoring”, “water demand”, “leak detection”, and “pipeline leakage”.

Starting from this first selection criteria, the search strategy can be shortly described as follows: (i) use of the previously defined keyword strings in various databases; (ii) selection of only published English papers in scientific peer-reviewed journals and conference proceedings in the last 30 years (1992–2022); (iii) preferring technical papers over surveys or other review articles; (iv) review of a sufficient description of devices and technologies; (v) detailed analysis of each device and technology and final inclusion in the overview.

**Figure 1:**
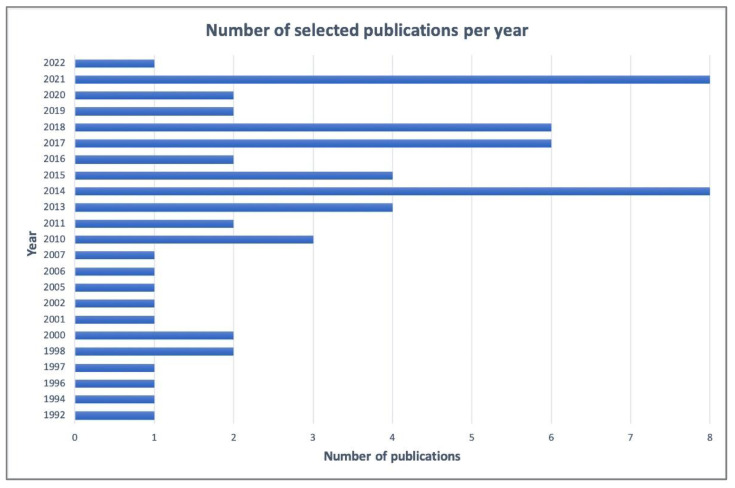
Number of selected publications per year (1992–2022).

**Figure 2:**
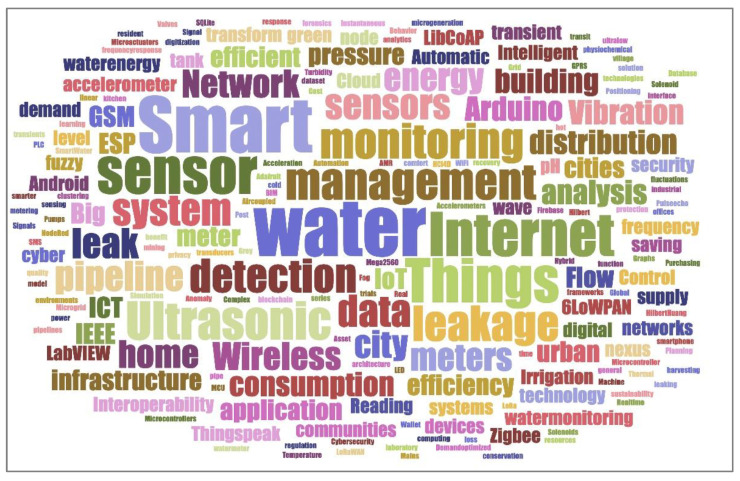
Graphical representation of the more frequent keywords in the selected publications.

Based on this search strategy, the overview was organized into three main sub-sections: water level, water consumption, and leak detection. Finally, 61 publications were selected (34 journal articles and 27 conference papers), published from 1992 to 2022, as reported in Figure 1, while the frequencies of the keywords in the selected publications are shown in Figure 2.

### 3.1. Water Level

Monitoring resource consumption plays a crucial role in intelligent water management. Overall, this is possible by installing water level measuring devices in a water tank or similar storage, whether in private or commercial applications. These measure devices generally consist of an emitter that generates the ultrasonic signal and a receiver element that receives the reflected wave [1]. The distance between the sensor position and the water surface is proportional to the delay between emission and reception of the ultrasonic wave packet [49].

In this regard, Min-Allah et al. [50] have developed an Android application based on the Internet of Things (IoT) for monitoring tanks’ water levels. The system works by using an ultrasonic sensor attached to the water tank that keeps track of the water level and which triggers an alert for the user if the water level is below a threshold level or empty. The architecture of this smart monitoring of water tanks system is divided into three main layers: (1) the physical layer (nodes and communication technologies that collect data and send them to the service layer); (2) the service layer (application/business logic, various tools for data analytics); (3) and presentation layer (visualizes the information to the user and allows user to interact with the system). In this proposed system, the physical layer is the physical environment (water source and associated ultrasonic sensor, which uploads data to the cloud server). The data can then be visualized via an Android application, which provides several widgets that update in real-time as soon as the ESP8266 Wi-Fi/Firebase is updated with the data stream.

While in the study [49], a tank height of 30 cm with an automatic motor to avoid water wastage is considered. If the water level becomes less than 20% of the tank height, the microcontroller sends a signal to the relay to turn on, and the motor starts. Instead, if the water level is greater than or equal to 80%, the relay automatically turns off and the engine also turns off. A message is sent to the user in both situations: in the first case, “Alert: level is 20%”, in the second case “, Alert: level is 80%”.

Raspberry technologies were also used by Sivaiah et al. [51]. The study suggests an IoT-based water monitoring system using an ultrasonic sensor. The measured level data are sent to the cloud thanks to Raspberry pi’s built-in Wi-Fi, and data readings are taken in real-time (every 20 s). The user can monitor the devices directly from the cell phone dashboard, viewing the water level.

Perumal et al. [52] proposed an IoT-based water monitoring system that measures the water level in real-time, applying IEEE802.11 communication standards and integrating a wireless gateway within the consumer network. The server collects the water monitoring data forwarded by the gateway, stores them in a database for analytics, and displays them in a web-based dashboard.

Dutta et al. [53] have presented a prototype of a smart building using technologies such as IoT, fog, and cloud. The idea is to measure the distance of the water level’s upper layer from the sensor attached point. When the tank water level becomes less than a threshold value, sensor HCSR04 automatically triggers the pump switch, sending water to the tank. After reaching the maximum level (threshold), the switch is turned off automatically. Using a fog server reduces internet traffic and makes the system more agile and responsive.

Shah et al. [54] have investigated a water level monitoring and control system with IoT and Android applications. Microcontroller ESP8266 obtains the maximum and minimum level values through the Firebase cloud. Ultrasonic sensors are used, and based on the water levels, the motor status is controlled by the android application, which shows the percentage of current water status instantaneous value. The use of ESP and an ultrasonic sensor have reduced cost-effectively and made the proposed project economical.

Praveen et al. [55] have addressed the household water overflow problem. The primary objective of the proposed model is to monitor the water tank level and sump visually. This research utilizes an ultrasonic sensor which is connected to an ESP module. The entire code is integrated with an Arduino IDE, and the real-time flow is displayed graphically via Adafruit. The proposed system helps the users to save water and understand their consumption rate.

In another study, Khan et al. [56] have shown a solution for water shortages in Saudi Arabia by developing a water monitoring system for customers. The prototype consists of two parts: the first is a website to display statistical information, enabling decision makers to implement more efficient water distribution policies and for customers to monitor their consumption rate and alert them to leaks. The second part consists of the hardware that detects the level and sends readings to the server for presentation on the website. The system has an ultrasonic sensor installed in the water container, to which a microcontroller is connected.

While in [57], a system was developed to monitor water use, prevent overflow, and find ways to save water. An ultrasonic sensor (HCSR04) measures reservoir level, while a flow sensor (YF-S201) measures water flow rate and total consumed volume. The level, flow rate and volume data are sent from the node MCU to the mobile application using the message queuing telemetry transport protocol (MQTT). Node MCU has been programmed by using Arduino IDE. Moreover, it is possible to control the solenoid valve operating as a tap from the mobile application.

Veselinović et al. [58] developed a system to save water in the toilet tank based on an ATMega 328 microcontroller. An ultrasonic sensor, water overflow sensor, and flush tank sensor are used for providing information to the microcontroller. The authors propose to add a Wi-Fi module and implement wireless communication between the device and user application as future progress.

In another study [59], the authors proposed a water-saving tool that automatically turns the water tap on or off using Arduino Uno with an ultrasonic sensor (HC-SRF04) and fuzzy logic algorithm to make the decision. The findings obtained during the experiment showed that water saving could be up to 70% per day, with beneficial economic effects for users and water providers.

Khan et al. [60] have presented an intelligent water level measurement using an Arduino Mega 2560 microcontroller, an ultrasonic sensor to detect the water tank level, a flow-meter, a Wi-Fi module, a display, and a pumping unit. The system saves inadequate water and electrical consumption by turning the pump unit on or off based on the water level.

Olisa et al. [61] have developed a system to monitor water quality and level in a two-tank water system. A pulse-echo ultrasonic technique (HCSR04 ultrasonic sensors) was used to control the water level. Other system components are a microcontroller (ESP32 with an integrated Wi-Fi module and Bluetooth module for wireless communication), actuator, electronic control valves, water pump, turbidity sensor, and pH sensor. With an Android mobile application, the user can monitor the level and quality of water in the overhead tank.

Table 1 presents a summary of the main components considered in the investigated systems for smart water level monitoring.

### 3.2. Water Consumption

In recent years, automated, real-time monitoring systems have been developed for intelligent management of water consumption and properly quantifying it. One method to manage consumption involves smart water meters, based on the Internet of Things (IoT) and cloud computing, equipped with specific algorithms to distinguish between normal and excessive water use. This raises users’ awareness of water use and promotes sustainable water management.

The system proposed by Khan et al. [56], whose components have been discussed in Section 3.3, allows homeowners to monitor their daily usage by providing illustrations for each water tap separately, as well as notifying the homeowner of a leakage possibility. In the system, all sensors are connected to a microcontroller to estimate consumption rate, leak detection, and tank level sent through the Ethernet shield to the website. This system can reduce shortages by driving water pumping to the most needed districts and reducing water waste since around 40% of water can be stored.

Santos et al. [62] have developed an android application to monitor power and water volume data that tenants and landlords can view. The meter devices read and measure the values during water and energy consumption, then process and combine the equivalent values. Thus, the data—stored in memory—is sent to the users’ smartphones to display the consumption. From the evaluations, the device is reliable, accurate, functional, and user-friendly.

In the study [63], a smart water meter system is developed, where the flow sensor reading is noted by NodeMCU and transmitted to the ThingSpeak Cloud platform. Machine learning is used to identify whether the flow pattern is normal, excessive, or continuous. When excess water flow occurs, the systems generate alerts sent to the users by email.

Vrsalovic et al. [64] have presented the development of a smart water meter IoT architecture. Axioma Qalcosonic water metering devices, equipped with LoRaWAN radio technology, measure the water consumption of some university building blocks. By observing the graphical representation of the average hourly water consumption, it is possible to detect uneven consumption that can indicate the presence of leaks in the system.

Suresh et al. [65] have proposed an innovative approach to perform automated water-meter reading and update consumption information. This approach differs from existing commercial methodologies, because it uses low-cost IoT hardware and a smartphone application. The approach was evaluated on a small section of the water distribution network of the Institute campus, saving around 14% of water thanks to corrective actions, replacements, and operations.

In another study, Tamilselvan et al. [66] developed a method to improve regular water delivery and control it from a central server to reduce water supply problems. The system uses an Arduino as a minicomputer, water flow sensor, and solenoid valves.

A wireless water consumption monitoring system whose main components are water flow rate/temperature sensors to collect data, which are routed to a remote computer server by home Wi-Fi and Internet, was designed by [67].

De Paula et al. [68] have proposed a smart system for remote monitoring of water consumption, detecting leakages and interruptions in water distribution. The hydraulic tier consists of two transducers (the water flow sensor and the pressure sensor) and one solenoid valve if some problems occur in the system. Specifically, the water flow transducer is a hall effect sensor with a digital output which can detect water flow between 1 and 30 L/min, with a maximum pressure of 1.75 MPa; by counting the number of pulses per minute, it is possible to obtain the measurement of flow and water consumption. At the same time, the pressure sensor can measure pressures in the range of 0 to 1.2 MPa. The electronic tier consists of two microcontrollers (MCUs): the MSP430G2553 MCU processes the acquired data and sends them by a UART channel to the second MCU (TI CC2650), which transfers the information to the In. IoT middleware uses a border router with Internet connectivity.

In another study [69], the FIKWater dataset is presented. The system consists of an ultrasonic flow meter (TUF2000M) installed in the main entrance pipe; the recorded data are sent by Modbus protocol to a local gateway, and then every minute data are uploaded by a standard HTTPS protocol to an online database server; finally, a CSV file with the daily readings is uploaded to a shared folder. The ultrasonic flow meter measures: instantaneous flow rate, liquid velocity, sound velocity, positive and negative accumulators, and totals (day, month, and year). The dataset contains time series of both hot- and cold-water demand data from three restaurant kitchens in Portugal, collected for consecutive periods between two and four weeks.

The study [70] presents a water management system based on wireless sensor networks (WSNs), consisting of three major components: the meter interface node, the gateway device, and the back-end system. With a web-based interface, it shows real-time and historical water consumption data. Therefore, the monitoring system can help users to reduce water consumption and identify possible leakage.

The study [71] proposed a fuzzy clustering algorithm to analyze residential water consumption patterns using smart meters.

In order to support decisions at both household and utility levels, Savica et al. [72] have created the iWIDGET system, which analyses the usage pattern of individual households, offering suggestions on how to reduce usage and take advantage of current tariff schemes, as well as sending an alert if local leakage is suspected. Therefore, by ICT, the system supports integrated water management, improving water use efficiency, reducing wastage by households, and enabling utilities to manage household water demand better.

Finally, another interesting study—although there are not many details about the IoT system—was carried out by Sodkomkham et al. [73]. It presents an integrated lean management, IoT, and MFCA system for achieving optimal water management in industrial production in Thailand. Water sensors to detect the amount and quality of water are used, but there are not many details about the other hardware and software components. In the case study, inputs, processes, and outputs were analyzed to reduce water use by recycling wastewater in processes and by applying the 3R (reduce, reuse, recycle) method. The results demonstrated a reduction in water use in production processes by 15% per year.

Table 2 shows the main components considered in the analyzed smart water consumption monitoring systems, excepting those of studies [71,72,73], since few details are reported, and study [56] since it was already shown in Table 1.

### 3.3. Leakage Detection

Water losses frequently occur in the city water distribution network [74]. Thus, leakage prevention and break identification are crucial to limiting water wastage. In this regard, to prevent water losses and decrease public risks, many techniques have been proposed with diverse applications for detecting the occurrence and sizes of leakage in water pipeline systems [75]. These devices—often connected to the network—can transmit information in real-time, control all of the data, and detect irregular water consumption and alarms related to leaks and micro leaks. Through these technologies, the whole building’s entire water system is effectively managed, with the possibility—for example—of water remotely shutting off or automatically shutting off in case of leaks to limit damage from uncontrolled water spillage into the building.

Thus far, several leak detection methods have been presented, and as reported in [76], they can be classified into three main categories: (i) biological methods—i.e., conventional leak detection methods based on individuals’ experiences; (ii) software-based methods—which use different computer software to detect leaks in a pipeline; (iii) hardware-based methods—which consider the use of different hardware devices to detect and locate the leak. The software-based method can be distinguished into flow or pressure change, mass or volume balance, dynamic model-based system, and pressure point analysis. The hardware-based methods can be classified into four types according to the principles of devices: visual devices, acoustic devices, gas sampling devices, and pressure wave detectors.

Based on this main classification, several studies that proposed software-based methods were found in the literature [77,78,79,80,81,82,83,84,85,86]. While volume balance method, as reported in [87], is one of the earliest computer methods developed. Moreover, as discussed in [88], for the continuous monitoring of a pipeline, another method is ATMOS PIPE which is a statistical pipeline leak detection technique. In another study, Salam et al. [89] developed a computerized online system to detect leakage with pressure analysis obtained from the EPANET software using the radial basis function neural network method.

Similarly, in the literature, several hardware-based methods were found. The majority of the studies have considered acoustic methods or methods based on vibration [90,91,92,93,94,95,96,97,98,99,100,101,102,103,104]. The studies [76,88] have considered visual observation methods, while ground-penetrating radar (GPR) methods were used in [105].

The researchers have shown a strong interest in vibration techniques for leak detection, considering accelerometers or ultrasound to measure the vibration signal of the water pipe [103]. In this regard, ref. [98] proposed a model for a real-time monitoring system based on wireless accelerometers. The accelerometers are installed on the exterior of the pipelines. Analysis of the vibration signal of each accelerometer sensor was assessed to determine the monitoring index. The collected data from the investigation were evaluated using the support vector machines (SVM) technique. A leak threshold was determined such that if the signal increased above the threshold, a leak status was identified. Experiments were performed on one-inch cast iron pipelines and one-inch and two-inch PVC pipelines using single event leaks, and the results were displayed. The developed models showed promising results with 98.25% accuracy in distinguishing between leak and non-leak states.

Almeida et al. [100] have investigated the combined filtering effects of the sensors (hydrophones, geophones, and accelerometers) and pipes for leak detection. The results showed that all three sensors could detect and locate a strong leak; on the other hand, a weak leak was not detected by the hydrophone sensors since there was noise in a narrow range of low frequencies—not related to the leak—that dominated the signals.

Study [103] proposed a non-attached ultrasound PVC pipe monitoring system. The system, which works with V-type ultrasonic air-coupled ultrasonic transducers, was tested in the laboratory. A pair of MA40S4R emitting ultrasound toward the pipeline were considered. The laboratory tests showed efficiency in recognizing leakages up to 0.4 mm in diameter with an accuracy of 94.97%, even for water pressure (less than 1 bar).

Dewi et al. [91] show the difference between output data from the normal pipe and pipe with leakage: the normal pipe produces more vibration than the pipe with leakage.

While in the study [106], a new interpretation of the cross-correlation process for estimating time delay in a vibroacoustic system is presented. Test data recorded from a specially constructed leak-detection facility located at the National Research Council in Canada were considered for validation. The results showed little difference in determining the time delay between the leak signals in the time domain using cross-correlation (GCC) methods or in the frequency domain using generalized phase spectrum (GPS) methods.

An interesting application is also the indirect approach to the problem, accomplished through pressure and temperature measurement and monitoring, which can help locate leaks. In this regard, Sadeghioon et al. [107] have designed and constructed a sensor for real-time leakage detection in pipelines based on measuring indirect relative pressure and temperature changes in plastic pipes. During the measurement campaign, the relative pressure and temperature sensors recorded leak tests and daily pressure changes.

A microelectromechanical systems (MEMS)-based wireless sensor network was developed by [93]. The system incorporates MEMS accelerometers for measuring flow-induced vibration on the surface of a pipe to evaluate the water pressure change due to leakage. Therefore, this study proposed using accelerometers attached to the pipe’s external surface instead of pressure sensors, whose installation is generally invasive. Thereby, this method presented a low-cost, nonintrusive pipeline monitoring system.

Daadoo et al. [108] focused on wireless sensor network applications for leakage detection in underground water pipelines. The wireless network system uses wireless mobile sensors that can detect breaks and save energy, time, and cost by smart water leakage detection (SWLD) in pipelines, measuring tank water level and controlling the pump to activate it when the water level is low.

Almazyad et al. [109] explored the use of mobile sensors by showing a simulation of a water pipeline monitoring system for leakage detection using radio frequency identification (RFID) and wireless sensor networks (WSNs); the system is for long-distance surface water pipelines.

The study [110] presents the development of a water pipeline monitoring system over ZigBee networks. The system’s main components are 6DOF MPU6050 sensors to collect the vibration data, Arduino UNO controller board, XBee module, and decision support system. The data are analyzed offline to define the conditions of the water pipeline.

Generally, the methods for leakage detection are either expensive, unscalable, or feasible only in the short term. Thus, Hester et al. [104] presented using environmentally powered embedded adaptive sensors to provide cost-effective water-monitoring infrastructure.

Most studies have analyzed monitoring systems for leakage detection for long-range pipes, while [90] presented a method of monitoring the condition of domestic pipelines. The system considered the operation of acoustic sensors and a couple of transducers (a transmitter and receiver). This approach is cheap and easy to install and maintain for homes and most other public facilities.

Britton et al. [111] have provided smart metering technology for households, allowing them access to water loss information. The residential leakage communication strategy has reduced minimum nighttime flows by a significant 89% throughout the study, while the group that received no communication increased consumption by 52%.

Kawarkhe et al. [49] designed a leakage detection system for pipes using two flow sensors. If the flow measured by the first sensor and the second one is not equal, a leakage is detected; thus, the GSM sends the message to the user.

Veselinovic et al. [58] have considered EPA data on average household leaks and the most common types of leaks. This study developed a smart home system to save water in the toilet tank, using a controller that opens and closes the valve and detects water leaks and toilet overflow. When a leakage or overflow is detected, the device performs the corresponding operation and indicates the type of error by turning on the specific LED (light-emitting diode). This paper explains water level measurement, leak detection, as well as the implementation of the device.

De Paula et al. [68] have proposed a solution in which all smart devices can communicate with each other, making everyone aware of the leakage problem. To perform the measurements, they have proposed to use two transducers (a water flow sensor and a pressure sensor) and a solenoid valve to stop the leaks as soon as they are detected. In a building, the water flow transducer must be installed near to the building water box, while the pressure transducer must be near to the hydraulic devices (shower, washing machine, or tap). Thus, if the system detects any abnormality, a relay activates the solenoid valve, and the water flow to the hydraulic devices is stopped.

As it emerged from the analysis of the selected studies, several leakage identification applications were performed for large-scale systems (urban drainage networks); some have presented monitoring system prototypes that can be used for different scales, while others—as already discussed in the previous sections—have analyzed the leakage detection in the building. Given the wide range of applications in this field and the different discussed methods, in Table 3, the main components of the system for leakage detection of 10 selected studies from those that have considered hardware-based methods are reported.

## 4. Smart Water Harvesting Systems

Increasing urbanization and climate change have impacted the natural water cycle with considerable effects in terms of increased runoff and flood hazards [112,113] and water scarcity phenomena [114,115]. These environmental impacts encourage widespread sustainable technologies, known as low-impact development systems (LID)—engineering techniques widely investigated in literature for their beneficial effects in reducing environmental impacts and obtaining proper water management in urban areas [116,117,118,119,120].

In this context, a promising strategy to save water and optimize its management is the implementation of water harvesting systems. These systems collect and reuse water from multiple sources (rainwater, greywater, atmospheric water). Thus, based on the type of water collected, these systems can be classified into rainwater harvesting systems and grey water harvesting systems.

The rainwater harvesting (RWH) system provides several benefits at different scales; it supplies decentralized water, manages stormwater, and increases local water security. This system collects rainwater from roofs, rooftop terraces, and impermeable surfaces to be reused on-site for different purposes, including irrigation of green roofs and gardens, flushing toilets, etc. Several studies have investigated the efficiency of these systems for water-saving and runoff mitigation [115,121].

Greywater is the wastewater collected from domestic washing operations—including showers, bathtubs, kitchen sinks, dishwashers, and washing machines; excluding blackwater sources (toilet, bidets, and urinals). Given its amount, this source can represent the most significant potential source of water savings for domestic use. Of course, given its low quality, treated greywater is mainly utilized for toilet flushing, garden watering, and car washing [122,123].

Although the conventional RWH systems are widespread, the advances in IoT technologies have affected their utilization, posing new optimization challenges. Thus, in the literature, smart water harvesting systems are gaining popularity.

In this regard, Maer et al. [124] proposed an original approach to classical reusable water collecting and reuse systems—i.e., rainwater harvesting systems (RHWs), drain water collecting, and wells—by automating the system using smart home technology. A reusable hybrid system combines available water sources at a specific location or time. This study emphasizes the most efficient and cost-effective solution to be implemented. The designated area of study is a household in Cluj-Napoca, Romania. The proposed water catchment and management system model was designed to be integrated, monitored, and controlled via smart home technology to use PV panels and low-consumption equipment. A proprietary web interface is used for automatic control and supervision. The authors used original approaches to reducing water scarcity by integrating environmentally friendly hardware equipment. This research aimed to provide an integrated smart home, tested, and functional solution for one of humanity’s biggest challenges—water scarcity.

Oberscher et al. [125] presented an innovative smart rain barrel integrated into a pilot project for smart cities, where weather forecasts and time-controlled filling levels of different low impact development (LID) structures and the connected sewer system are used for real-time control (RTC).

In another study, Behzadian et al. [126] investigated a smart RWH system that proactively controls the tank water level to guarantee sufficient storage to receive stormwater runoff. The analysis using the WaterMet2 model emerges that the smart system can significantly mitigate local flooding and supply harvested rainwater to non-potable residential water consumption.

In conclusion, a smart combination of sustainable systems and intelligent technologies can significantly improve water resource management and enhance sustainable development.

## 5. Discussion, Current Challenges, and Future Directions

Section 3 provided an overview of IoT-based smart water monitoring and control solutions, focusing primarily on systems that are useful at the building scale and then at the urban scale. From this overview, it emerges that the crucial components of an IoT-based water management system can be principally classified into hardware units and software units. As reported in [12], sensors, actuators, and smart meter devices—main hardware components—are used to collect data, and communication technology is used to connect the overall elements of the systems; while the cloud platform stores, processes, and analyses the data.

Based on this main classification, ultrasonic sensors are the most used in analyzing the studies on the systems developed to monitor water levels. This type of sensor generates ultrasonic sound waves bombarded on the water’s surface [54]. Among these devices, HCSR04—consisting of an ultrasonic transmitter, receiver, and control circuit—is commonly used for non-contact distance measurement from 2 cm to 400 cm [51]. Another sensor used in systems to monitor water consumption is the Hall-effect-based water flow sensor. Among these devices, the YF-S201 is one of the most used. This type of sensor has a pinwheel sensor to measure the amount of water moving through it, and it also has an integrated magnetic Hall-effect sensor that outputs an electrical pulse with every revolution [63]. As reported in [57], this sensor has an operating flow rate of 1 to 30 liters per minute. Smart water metering devices are widespread in the water consumption monitoring system. Finally, by analyzing the developed systems for leakage detection, accelerometers were the most used. In this regard, accelerometers have garnered researchers’ attention since they can be used as a complete leak detection system to detect vibration signals emitted by leaks [127].

Moreover, as reported in [15], the use of wireless sensor networks—considered in different studies of this overview—cannot be overlooked.

The control unit is generally the core hardware of all monitoring and control systems; from the review conducted here, the ESP Wi-Fi modules, Raspberry Pi, and Arduino in their different categories resulted in the most used. More in detail, ESP 8266 is a Wi-Fi module used as a microcontroller programmed to implement logic statements [54]. It comes with a powerful Wi-Fi module, which allows information transfer through Wi-Fi and consists of a storage capacity and powerful enough onboard mechanism to coordinate with the sensors [55]. It is cheaper than Raspberry Pi and other micro-controllers, making the system more affordable. Moreover, this microcontroller with specific sensors effectively reduces cost, providing flexible, economical, and easy configurable systems [57]. Raspberry Pi is configured as a credit-card-sized microcomputer based on a Raspbian Linux operating system with less complexity and more affordable solutions for wireless monitoring [128]. However, several of the investigated studies have used Arduino boards as a control unit and, more in detail, Arduino Uno [59,104], Arduino Pro [53], Arduino Mega2560 [60,108], and Arduino Ethernet SHIELD [66]. As reported in [129], Arduino Uno, which is based on ATmega328 microcontroller; and Arduino Pro, which can be based on ATmega168 or ATmega32, belong to the Arduino Boards category classified as Entry Level Board; while Arduino Mega 2560, which is based on ATmega2560, belongs the category of enhanced features boards. Entry level boards are most easy to use and program, while enhanced features boards are designed for complex project development and present more features and performance than entry-level boards. Finally, Arduino Ethernet Shield (Internet of Things (IoT)) facilities connectivity to Arduino board. Generally, all the Arduino Shields are specially designed for beginners to overcome the complexity of connecting components and to add more hardware resources. Arduino is widespread primarily due to its features: an independent platform, low cost compared to other microcontrollers, open source hardware and open source software, and ease of programming via Arduino IDE.

As it is possible to detect in Section 3, different communication technologies have been considered (Wi-Fi, Bluetooth, RFID, Zigbee, LoraWAn, Cellular network, etc.). Bluetooth and RFID (radio frequency identification) are wireless connectivity technologies with a short-distance communication range (at most 10 m); ZigBee and Wi-Fi operate within a medium distance range (10 to 100 m); while cellular networks (2G/3G/4G/5G), and low-power wide-area (LPWA) technologies (which includes LoRa) operate in the long-distance communication range category [130]. Among these technologies, ZigBee is a wireless communication technology operating on the IEE 802.15.4, which addresses the need for low-rate, low-power, and low-cost wireless networking [131]. In this regard, as reported in [132], it presents a low rate between 20 kbps and 250 kbps, compared to Wi-Fi’s 11 Mbps or Bluetooth’s 1 Mbps; network join times of around 30 ms, compared to Wi-Fi’s up to 3 s or Bluetooth’s up to 10 s; lower power consumption (ZigBee devices can operate for several years on a single battery); low cost of products and cheap implementation. Moreover, it can support hundreds of devices compared to Wi-Fi which can support up to 32 devices or Bluetooth which can support up to 7. Finally, LoRaWAN (LoRa wide area network) is a network based on LoRa technology, which is one of the most promising low-power wide-area communication technologies. It enables long-range transmission with low power consumption, and it can achieve data rates between 0.3 kbps and 27 kbps. How to implement a flexible LoRa network with an effective cost is still an open challenge [131].

Another crucial element in IoT-based systems is the cloud platform, which—together with big data platforms—is suitable for storing large-scale datasets through database management systems, preprocessing, statistical analysis, and data visualization [11]. These platforms perform logical analysis and complete control over the functionality of the IoT-enabled devices [13]. Some of these also provide apps that monitor and control the IoT elements by mobile devices [10]. Following the widespread of IoT devices, several IoT cloud platforms were developed, such as ThingSpeak, Blynk, Arduino Cloud IoT, IBM IoT, Adafruit, and others [13]; some of these were also considered in the manuscripts investigated in Section 3. In more detail, ThingSpeak Cloud Platform is an open IoT data platform that can easily configure devices to send data through standard IoT protocols and visualize the sensor data in real-time [63]. ThingSpeak IoT Platform and Arduino IoT Cloud allow data collection in private channels with free hosting for channels, app integration, and event scheduling [133]. The IBM Watson IoT Platform can be used for any IoT solution, including smart homes; it provides machine-learning services to adopt into IoT applications and data analytics [134], while Adafruit provides different statistical tools on single clicks [55]. Finally, as reported in [134], different Cloud infrastructures have been developed (OpenStack, OpenNebula, and CloudStack) to monitor, manage, and offer an infrastructure to deploy Cloud platforms. More in detail, OpenStack consists of a pluggable set of components, while the other two present a centralized architecture; therefore, OpenStack better meets the user’s needs but requires higher installation efforts than the others. To address communication requirements and scalability issues, Merlino et al. [135] proposed Stack4Things, an extension of the OpenStack platform to enable a cloud-oriented infrastructure for IoT management.

In Section 4, the use of smart technologies to optimize the use of sustainable solutions for water harvesting systems is analyzed. These studies show the enhanced efficiency of smartly equipped conventional harvesting systems.

Therefore, this overview identified the main components of the IoT monitoring and control of smart water management systems, and a comparison was also presented. However, through in-depth analysis, each technology presents advantages and limitations that should be overcome to increase the applicability of these systems starting from the building scale.

Cost of deployment, power usage, maintenance, privacy and security, connectivity coverage, complexity, and ease of operation are still open challenges. In this regard, the future development of low-cost devices with higher energy efficiency is crucial in supporting and driving widespread IoT applications in the water management sector. According to Khanh et al. [131], another aspect that will continue to be a research topic for academic and industry research in the future is privacy and security. As reported in [14], the amount of data collected in IoT-based systems—like those investigated in this review—is vast and vulnerable to cyber threats, especially during the transmission to the data server. Moreover, as previously discussed, the commonly used wireless communication protocol has some limitations concerning the power requirement of some technologies, as well as the communication distance they can cover [136].

Moreover, another major challenge is the perceived complexity of smart water systems related to installation, operation, and maintenance activities [63]—as well as the need to develop smart water management systems to be more adaptable and replicable in different contexts and locations [11].

In conclusion, based on all of this discussion, an in-depth analysis of the investigated articles supports the readers in identifying the main challenges, relevant recommendations, and future directions for IoT applications for smart water management.

## 6. Conclusions

Increased population and industrial activities combined with climate change present a serious issue regarding water resource availability. The water scarcity phenomenon is increasing and represents one of the global environmental impacts. Thus, monitoring water usage and adequately managing this limited resource is one of the main aims of researchers in recent years. Proper water management can optimize the use of this resource by reducing waste and managing supply. Several studies have investigated the main technologies to reduce water wastage. Most of them focus on the smart management of this resource through IoT advanced technologies.

This paper presents an overview of the innovative systems for smart water resource management. Specifically, we focused on the innovative technologies to monitor, control, and manage water levels, water consumption, and water leakage starting from the building scale. Finally, innovative technologies were analyzed by combining sustainable systems—such as water harvesting systems—generally used to save and reuse water resources.

This comprehensive overview revealed the importance of investigating the potentiality of water-saving innovative and sustainable technologies to optimize resource management by limiting technical losses and human overuse.

## Figures and Tables

**Table 1:** Summary of the main components used in the investigated smart water level monitoring systems.

Ref.	Hardware Components	Software Components, Communication Technologies, Cloud Platforms, Decision Making
[49]	Ultrasonic sensor; microcontroller	Arduino IDE; GSM modem
[50]	Ultrasonic sensor; Arduino; ESP8266 Wi-Fi module	Firebase; Android Application
[51]	Ultrasonic sensor; Raspberry Pi	Wireless LAN and Bluetooth
[52]	Ultrasonic sensor; ATmega328P	IEEE802.11 communication standards; wireless gateway
[53]	Ultrasonic sensor HC SR 04; pump; Arduino Uno/Pro Mini (microcontroller board based on the ATmega328); Wi-Fi module-ESP8266; Relay	Bluetooth module-HC 05; fog gateway server; cloud platform
[54]	Ultrasonic sensor; ESP 8266 as microcontroller	Firebase cloud; Android application
[55]	Ultrasonic sensor HCSR04; motor; ESP8266 Wi-Fi module	Arduino IDE with C as the programming language; web application; Adaftruit
[56]	Ultrasonic sensor; water flow sensor; Arduino	Wi-Fi; website
[57]	Ultrasonic sensor HCSR04; YFS-201 flow sensor; 12V DC solenoid valve; Node MCU ESP8266	Arduino IDE; SQLite database; MQTT mosquito broker; Android application
[58]	Ultrasonic sensor HC-SR04; water overflow sensor; tank flush sensor; ATMega 328 microcontroller	Future idea: add a Wi-Fi module and implement wireless communication between the device and user application.
[59]	Ultrasonic sensor HC-SRF04; Arduino Uno	Fuzzy logic method or making decision; database
[60]	Ultrasonic sensor AG222VXCM0800US1; flow sensor (Hall-effect-based); motor; pump; Arduino Mega 2560 microcontroller; ESP8266 Wi-Fi module; relay	Wi-Fi; mobile application
[61]	Pulse-echo ultrasonic technique; ultrasonic sensor HCSR04; actuator and electric water pump; ESP32 Wi-Fi module and Bluetooth module	Firebase; Android mobile application; wireless communication

**Table 2:** Summary of the main components used in the investigated smart water consumption monitoring systems.

Ref.	Hardware Components	Software Components, Communication Technologies, Cloud Platforms, Decision Making
[62]	Water meters; wattmeter	Wi-Fi; Android Application
[63]	YF-S201 water flow sensor; NodeMCU	ThingSpeak Cloud platform; Machine Learning Tools
[64]	Axioma Qalcosonic water metering device (with ultrasonic technology)	Semtech SX1301/1257 LoRaWAN technology; Things Network (TTN) cloud infrastructure
[65]	Water-meter Hall-effect-based sensor; electronic interface module—EIM (designed around an Arduino SBC board with Ethernet stack and additional flash memory)	TCP/IP network or Wi-Fi or Bluetooth or GSM/3G/4G router or Optic-fiber; smartphone App
[66]	YF-S201 water flow hall effect sensor; solenoid valves; relay circuit; Arduino Ethernet SHIELD V1	Mobile phone application
[67]	Water flow rate/temperature sensor	Local wireless monitoring unit (wireless data collectors, Wi-Fi router and Wi-Fi gateway); remote central server; home Wi-Fi network and Internet; remote server software (Visual Studio 2012 and Microsoft SQL server 2014)
[68]	YF-S403 water flow hall effect sensor; Seeed Studio water pressure sensor; solenoid valve; two microcontroller units—MCUs (MSP430G2553 and TI CC2650); universal asynchronous receiver/transmitter (UART) protocol	Standard IEEE 802.15.4; border router; In.IoT middleware; wireless communication; MQTT protocol
[69]	TUF2000M ultrasonic flow meter; Raspberry Pi; transducers; 3S battery	Modbus protocol; gateway; standard HTTPS protocol; online database (JSON); shared folder (CSV)
[70]	Wireless meter interface sensor node; digital water meter; analogue Reed switch; Dizic module (STM32W108 processor with an integrated 2.4 GHz transceiver); Rasberry Pi; serial flash (AT25DF321); MCP73871 microcontroller (for charging system); buck-boost converter (TPS63001)	IEEE 802.15.4; ZigBee supporting network; Contiki OS; firmware; LibCOAP; 6LowPAN (IPv6 over Low Power Wireless Area Network); 802.11 and 802.15.4 communication interface; wireless communication; Pandora Flexible Monitoring Software (FMS) agent; database; visualization engine; web interface

**Table 3:** Summary of the main components used in some hardware-based investigated systems for smart leakage detection.

Ref.	Hardware Components	Software Components, Communication Technology Cloud Platforms, Decision Making
[90]	Acoustic sensors; ultrasonic transmitter transducer; ultrasonic receiver transducer; Arduino microcontroller	Wi-Fi; LabVIEW software
[100]	Hydrophones 8103 by Bruel and Kjaer, geophones SM-24 by Ion; accelerometers 4383 and 4384 by Bruel and Kjaer; Charge Amplifiers 2635 by Bruel and Kjaer	DATS (Acquisition System by Prosig)
[102]	Vibration Sensor MPU6050; pressure meter	iMote; GPRS/ZigBee wireless; decision support system; Mobile
[103]	V-type air coupled ultrasonic transducer—MA40S4R;	LCD; wireless
[104]	2 Arduino Uno; Minisense 100 piezo sensor; ADXL335 accelerometer	10-port ethernet switch; fiber jumper; standard desktop computer with Ethernet connection as a data collection server
[91]	Vibration sensors MMA7361 k; Arduino controller board	Wireless XBee Pro Module transmitter; Wireless XBee Pro Module receiver; X-CTU software; decision support system; mobile phone
[93]	Accelerometer (MMA7361 Model: 1156); Liquid flow sensor; PIC18F2620 microcontroller; KYL-500S Transceiver; 9V battery terminal for mobile operation; LM7805CV voltage regulator; RS232 serial port connector for connection to PC COM port	Operating system software (firmware); Scalable Interdomain Routing Addressing Scheme (SIRAS); KYL500S radio; wireless
[108]	Water sensors; ultrasonic sensor; Arduino Mega 2560 microcontroller; water pump; solenoid valve; relay 12v	Arduino IDE; GSM modem; wireless; Android mobile application
[109]	Mobile wireless sensor node with pressure sensor (Intersema MS5541C); low-energy microcontroller (LPC1102 Cortex-M0);	RFID reader (Tagsense ZR-232 Active Tag Reader); RFID tag (Tagsense ZT-50 Active RFID Tag); Wireless technology
[110]	Accelerometer sensor MPU6050 with water pressure and a flow rate meter; Arduino controller board	Wireless ZigBee Pro module (transceiver module); decision support system; mobile phone.
